# Evaluation of procalcitonin as a marker of bacterial sepsis in children in Owerri, Nigeria

**DOI:** 10.4314/ahs.v25i4.2

**Published:** 2025-12

**Authors:** Ogechi Ezerioha, Emeka Nwolisa, Joseph Ezeogu, Udochikwuka Ikejiaku

**Affiliations:** Department of Paediatrics. Federal Teaching Hospital Owerri, Nigeria

**Keywords:** Infection, Children

## Abstract

**Background:**

Paediatric sepsis is a significant cause of morbidity and mortality, particularly in low- and middle-income countries. Timely diagnosis remains a challenge due to the limitations of conventional blood culture. This study evaluates procalcitonin as a biomarker for bacterial sepsis in children aged 0–59 months in a resource-limited setting.

**Methods:**

This cross-sectional study was carried out between June 2023 and February 2024. It involved 298 children aged 0–59 months with systemic inflammatory response syndrome (SIRS) who were admitted to the Emergency Pediatric Unit and Special Care Baby Unit of Federal Teaching Hospital, Owerri. Serum procalcitonin levels were measured using ELISA, and blood cultures were processed using the BacT/Alert system. Sensitivity, specificity, and predictive values of procalcitonin were compared against blood culture results.

**Results:**

Of the 298 children, 142 (47.7%) had culture-proven sepsis. The sensitivity, specificity, positive predictive value, and negative predictive value of serum procalcitonin were 66.9%, 89.7%, 85.6%, and 74.9%, respectively. The area under the curve (AUC) for serum procalcitonin was 0.80 across all age groups which were statistically significant.

**Conclusions:**

Procalcitonin has a good diagnostic accuracy in detecting bacterial sepsis in children and can complement blood culture in resource-limited settings. Further studies are recommended to explore its application in routine clinical practice.

## Introduction

Paediatric sepsis remains a major healthcare problem affecting millions of children with high morbidity and mortality, especially in sub-Saharan Africa. In low- and middle-income countries, children under the age of 5 years are 18 times more likely to die than those in higher-income countries partly due to the inability to provide timely and appropriate medical services^1^.

The 2015 Sepsis Prevalence, Outcomes, and Therapy (SPROUT) study, involving 128 pediatric intensive care units (PICUs) across 26 countries, reported a sepsis point prevalence of 8.2%, with an overall mortality of 25%. Mortality varied by region with the highest observed in Africa (23.1%)^2^. Furthermore, in 2017, it was estimated that nearly half of global sepsis cases occurred among children, with approximately 20 million cases and 2.9 million deaths in children under five years of age^3^.

Nigeria remains a significant contributor to global under-five mortality, with sepsis identified as a major cause^4^. Studies in various parts of Nigeria, including Zamfara^5^, Akure^6^ and Nnewi^7^ have consistently reported sepsis as a leading cause of mortality among under-five children admitted to Emergency Paediatric Unit. Delayed diagnosis, often reliant on blood culture as the gold standard, exacerbates this issue.

Blood culture remains the first-line tool in the diagnosis of sepsis, however, because most resource-poor countries like Nigeria make use of the conventional blood culture system rather than the automated type owing to cost and unavailability; blood culture result is often delayed. Also, the automated blood culture system can yield positive bacterial growth within 12-16 hours unlike the conventional blood culture method that takes 24-72 hours to detect bacterial growth^8^. This leads to making a diagnosis, based on the clinical features of sepsis which are generally non-specific and tend to overlap with other non-infectious causes of systemic inflammation. This also promotes unnecessary commencement of empirical antibiotics at presentation. This inaccurate and unnecessary use of antibiotics can cause adverse reactions while being a major cause of increasing rates of antibiotic resistance.

As the incidence of bacterial drug resistance continues to increase across the globe, it becomes important that diagnosis of bacterial sepsis is made early to enhance antibiotic stewardship. An ideal biomarker would help to make an early diagnosis, facilitate therapeutic interventions and decisions, and aid the recovery of patients.

Procalcitonin (PCT) has emerged as a promising biomarker for the early and accurate identification of bacterial infections. PCT is secreted by neuroendocrine cells of the lungs and intestines in response to bacterial infections. It increases 2 – 4 hours post-bacterial infection and can be detectable for up to seven days. There are no enzymes in the plasma that break down PCT. Therefore, if PCT enters the circulation, it remains unchanged, with a half-life of 22 – 26 hours^9^. Under physiologic state, very low serum PCT levels (< 0.05 ng/mL) occurs. However, the synthesis of PCT can be increased up to 100 to 1,000-fold in bacterial infections as a result of endotoxin and/or cytokines like interleukin (IL)-6, tumour necrosis factor (TNF)-alpha and IL-1b, which act on various tissues. In contrast, cytokines, such as interferon (INF)-gamma, which is released following viral infection, lead to down-regulation of PCT which makes it attractive as a potential diagnostic variable for the diagnosis of bacterial infection^10^.

Procalcitonin also exhibits greater sensitivity and specificity than other proinflammatory markers such as C-reactive protein (CRP) as a screening marker for sepsis when used in conjunction with other diagnostic tools and clinical examination^11^. This was demonstrated by Simhachalam et al.^11^ who studied the usefulness of different biomarkers in diagnosing bacterial sepsis in children. The study showed that PCT has a higher sensitivity and specificity (90.3%, 92.9%) compared to CRP (87.5%, 57.1%) respectively. In Ghana, Amponsah et al^12^ also reported that PCT was a better predictive marker for neonatal sepsis than CRP with sensitivity (87.5%) and specificity (63%) in relation to CRP (50%, 72.2%) respectively.

## Method

This cross-sectional study was conducted at the Emergency Paediatric Unit and Special Care Baby Unit of the Federal Teaching Hospital, Owerri, (FUTHO) Imo State, Nigeria. The hospital is a tertiary facility in the South Eastern region of Nigeria which serves as a referral center for neighboring states.

### Recruitment of participants

Three-hundred children were recruited consecutively until the desired sample size was met between June 2023 and February 2024. Children aged 0-59 months with evidence of Systemic Inflammatory Response Syndrome (with at least two of the following):^13^

Temperature > 38.5°C or < 36°C (Axillary).
Tachycardia >130 beats per minute for neonates and infants and >100 beats per minute for 1 year and above.
Respiratory rate > 50 cycles per minute for neonates and infants and > 30 cycles per minute for 1 year and above were included in the study.
Leucocyte count elevated or depressed for age or >10% immature neutrophils.
Exclusion criteria included children with severe burns, trauma, and surgery; Fever that has lasted more than 1 week (PCT is detectable for up to 7 days). Data on sociodemographic characteristics and clinical features were collected using structured questionnaires. A thorough physical examination was performed on each child. Each child's length/height, weight, temperature, pulse/heart rate, and respiratory rate was taken and documented in the proforma.

### Sample collection and processing

#### Blood culture

The blood culture technique used in this study was the automated detection system. Two milliliters of blood was taken under strict aseptic conditions and dispensed into the BacT/Alert® PF Plus culture bottle (BioMerieux, Inc., Durham, USA). The samples were transported to a microbiology laboratory immediately after collection. Samples with growth were sub-cultured on blood agar, chocolate agar and MacConkey agar plates and organisms were further identified by the Microbact 24E oxoid identification system.

#### Procalcitonin

The specimen for PCT was serum and taken with the usual precautions in the collection of venepuncture samples. The blood was collected in redtop venepuncture tubes with no anti-coagulants. The blood was allowed to clot for serum samples. The specimens were centrifuged to separate the serum from the cells and assayed using Enzyme-linked Immunosorbent assay (ELISA) quantitatively based on antigen-antibody reactions. The ELISA kit was that of Accubine® and was stored under optimal conditions determined by the manufacturer and shelf life.

#### Data Analysis

The data were analysed using the Statistical Product and Service Solutions by IBM (IBM SPSS, Chicago, IL, USA), version 24.0 running on MacOS. The data were presented in frequency tables, charts and summary statistics. The Kolmogorov-Smirnov test was used to determine the normality or otherwise of the serum PCT levels and other quantitative data. Parametric data such as temperature, and heart rate were summarised as mean and standard deviations (SD), while skewed data such as PCT levels, age, respiratory rate, weight and height were expressed as medians and interquartile ranges (IQR). The sensitivity, specificity, and positive and negative predictive values of serum PCT were calculated against the blood culture result to assess its diagnostic performance as a marker of sepsis. The PCT levels of the children with positive or negative blood culture results were compared using a contingency table. In children with positive blood culture results, the median PCT values were compared among the types of organisms isolated, whether gram–positive or gram–negative. The Mann-Whitney test was used to determine the difference in the median between the two groups. The Chi-square test was used to determine associations between categories (age groups, demographics, presence or absence of sepsis, etc.). A 95% confidence interval was used throughout the study and a p-value < 0.05 was considered significant. The receiver operative characteristic curve was plotted for PCT and AUC was calculated at a cut–off of 2.0 ng/mL.

## Ethical approval

Ethical clearance was sought and obtained from the Research and Ethics Committee of the Federal Teaching Hospital, Owerri. Parents and legal guardians of eligible children were asked to provide written informed consent.

## Results

### Sociodemographic Characteristics of Study Participants

A total of three hundred children aged between 0 – 59 months were recruited for the study. However, 298 samples were eventually analysed because 2 blood culture samples were contaminated owing to isolation of skin commensals. The classification of age was based on stages of development: Neonates, infants, toddlers, and preschoolers. The median age of the study participants was 9 months and the male – female ratio was 2.1:1. Socioeconomic classification was determined using the scheme proposed by Oyedeji.14 Most of the participants (32.6%) were in the age group 29 days – 12 months. The participants were mainly from the middle socioeconomic class (47.0%) as shown in Table 1.

**Table 1:** Sociodemographic Characteristics of Study Participants

Sociodemographic variables	n = 298	
	Frequency (%)	
**Gender**		
Female	97	32.6
Male	201	67.4
**Median age in months** (*interquartile range*)	9	(1 – 24)
**Age interval**		
0 - 28 days	75	25.2
29 days – 12 months	97	32.6
13 – 24 months	62	20.8
25 – 59 months	64	21.5
**Socioeconomic class**		
Upper class	79	26.5
Middle class	140	47.0
Lower class	79	26.5

### Procalcitonin Levels in Study Participants with Culture - positive or negative Sepsis

Among the 298 children, 136 (45.6%) had serum procalcitonin (PCT) levels below 0.5 ng/mL, 51 (17.1%) had PCT 0.5 to < 2.0 ng/mL, and 111 (37.3%) patients had serum PCT ≥ 2.0 ng/mL.

**Figure 1:**
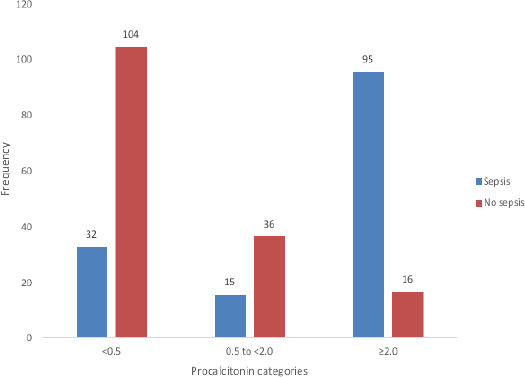
Procalcitonin Levels in Study Participants with Culture - positive or negative Sepsis

Out of the 111 children with PCT level 2.0 ng/mL, majority (85.6%) of them had culture-positive sepsis while only 23.5% of those with PCT level < 0.5 ng/mL had culture-positive sepsis (Figure 1).

### Contingency Table of Procalcitonin and Blood Culture Results in Study Participants

More of the children with culture – positive sepsis have elevated PCT ≥ 2.0 ng/mL (95, 31.9%) when compared to those with culture – negative sepsis (16, 5.4%). On the other hand, more children with culture – negative sepsis have PCT lower than 2.0 ng/mL (140, 47.0%) when compared with children who had culture – positive sepsis (47, 15.8%). This is depicted in Table 2.

**Table 2:** Contingency Table of Procalcitonin and Blood Culture Results in Study Participants

		Blood culture		
		Positive	Negative	Total
Procalcitonin	**Positive**	95 (TP)	16 (FP)	111
	**Negative**	47 (FN)	140 (TN)	187
	**Total**	142	156	298

### Diagnostic Indices of Procalcitonin in the Study

The sensitivity of PCT when compared to blood culture in predicting bacterial sepsis was 66.90% (true positive) while the specificity was 89.74% (true negative), with positive and negative predictive values of 85.59% and 74.87% respectively. The accuracy of PCT in this study was 78.86% (Table 3).

**Table 3:** Diagnostic Indices of Procalcitonin in the Study

Diagnostic Index		Value	(95% Confidence Interval)
Sensitivity	(95/142)	66.90%	(58.52% – 74.56%)
Specificity	(140/156)	89.74%	(83.88% – 94.02%)
Positive Predictive Value	(95/111)	85.59%	(78.63% – 90.55%)
Negative Predictive Value	(140/187)	74.87%	(70.09% – 79.11%)
Positive Likelihood Ratio	((95/142)/(16/156))	6.52	(4.04 – 10.52)
Negative Likelihood Ratio	((47/142)/(140/156))	0.37	(0.29 – 0.47)
Accuracy	((140+95)/298)	78.86%	(73.78% – 83.36%)

### Procalcitonin Receiver Operating Curve (ROC) for Sepsis

Overall, the area under the curve (AUC) was 0.80 with a standard error (SE) of 0.027 and this was statistically significant with p-value < 0.001. The AUC was higher in participants aged 0 – 28 days and 13 – 24 months with values of 0.899 and 0.847 respectively (Table 4).

**Table 4:** Procalcitonin Receiver Operating Curve for Sepsis

	AUC	Standard error	95% Confidence Interval	p-value
0 – 28 days	0.899	0.035	0.831 – 0.967	<0.001
29 days - 12 months	0.726	0.056	0.616 – 0.836	<0.001
13 – 24 months	0.847	0.054	0.740 – 0.954	<0.001
25 – 59 months	0.716	0.067	0.585 – 0.848	0.003
***Overall***	***0.80***	***0.027***	***0.748–0.852***	***<0.001***

Children that had positive blood cultures had median PCT levels of 2.22 ng/mL (IQR 0.33 to 6.05ng/mL) which was greater than those of children whose blood cultures were negative, and this was statistically significant (median procalcitonin level = 0.18 ng/mL (IQR 0.02 to 1.05 ng/mL); Mann-Whitney U z-score -8.96, p < 0.001). The PCT levels were more elevated in children from whom Gram – negative organisms were isolated compared to those from whom Gram – positive organisms were isolated, and this was statistically significant (median value (IQR): 4.68 (2.11 to 21.65 ng/mL) versus 2.14 (0.23 to 4.38 ng/mL); Mann-Whitney U z-score -4.44, p < 0.001).

## Discussion

This study sought to evaluate the diagnostic performance of serum PCT as a marker of bacterial sepsis in children aged 0 – 59 months seen at the Federal Teaching Hospital, Owerri.

The current study demonstrates that PCT levels were higher in children with culture-positive sepsis compared to those with culture-negative sepsis (31.90% versus 5.40%). These findings are consistent with studies conducted in the United States by Damman et al^15^ and Nellis et al.^16^ as well as research carried out by Matha et al.^17^ in Australia and Arowosegbe et al.^18^ in Abeokuta. These studies showed a significant increase in PCT levels in paediatric patients with bacterial sepsis, emphasizing its usefulness as a valuable marker. Studies carried out by Arowosegbe et al.^18^ Sakyi et al.^19^ Das et al.^20^ and Nath et al.^21^ in Abeokuta, Ghana, and India showed varying cutoff levels of 0.5 ng/mL, 1.57 ng/mL, 2 ng/mL, and 2.25 ng/mL, respectively, for the detection of bacterial sepsis. Although, all these studies used different cut–off levels for PCT, all the authors^15^,^18^,^19^,^20^ including the researcher in the current study have documented that paediatric patients with bacteraemia have increased PCT compared to children without bacteraemia.

In this current study, PCT showed sensitivity, specificity, positive predictive value and negative predictive values of 66.90%, 89.74%, 85.59%, and 74.87%, respectively. This is similar to a study by Das et al.^20^ who documented comparable values of sensitivity, specificity, PPV and NPV rates of 70%, 81%, 77% and 75% correspondingly at a cutoff of ≥ 2 ng/mL. However, Nellis et al.^16^ reported a sensitivity of 69.20% which is like the current study, but with lower specificity and PPV rates of 74.40% and 28.60%, and a higher NPV rate of 94.20%. The higher NPV rate documented by Nellis et al.^16^ may be because the study was retrospective, which is prone to selection bias and also, those with prior antibiotic exposure were excluded unlike the current study which was prospective and included children with prior antibiotic use. In addition, Nath et al.^21^ reported a comparable sensitivity of 65.12% with a lower specificity and PPV of 71.60% and 27.72% respectively at a cutoff of ≥ 2.25 ng/mL. However, the researchers reported a higher NPV of 92.47% compared to the 74.87% obtained in the current study. The higher PPV and lower NPV rate recorded in the current study may be attributed to the inclusion of all febrile patients who met the SIRS criteria in contrast to the study conducted by Nath et al.^21^ who recruited paediatric cancer patients with features of SIRS.

In Pakistan, Habib et al.^22^ reported sensitivity, specificity, PPV and NPV rates of 97.70%, 70.60%, 77.1%, and 96.80%, respectively. The higher sensitivity value recorded by Habib et al.^22^ may be accounted for by the fact that the study was done only in neonates and a lower cutoff level of PCT was used (0.5 ng/mL) compared to the present study which was carried out in underfive children and a higher PCT cutoff level was used. In contrast, Ezeh et al.^23^ in Calabar, recorded a comparable sensitivity rate of 68.40%, but with lower specificity, PPV and NPV rates of 29.30%, 31%, and 66.70%, respectively. Whereas, the current study recruited three-hundred children and used the BacT/Alert blood culture system, Ezeh et al.^23^ recruited 60 neonates with suspected sepsis and used the conventional blood culture method.

In the present study, the receiver operating characteristics curve analysis for PCT in predicting systemic bacterial infections across all age – groups showed area under the curve (AUC) of 0.80 which was statistically significant. This implies that PCT is a useful tool in early diagnosis of systemic bacterial infection. This agrees with Damman et al 15 who studied children and young adults aged 0 – 23 years with central line and fever but recorded a comparable AUC of 0.85 on ROC curve analysis. Additionally, Nellis et al, 16 Sakyi et al 19 and Nath et al.^21^ recorded AUC values of 0.790, 0.787, and 0.751, respectively. This further reaffirms the findings in the current study which shows that PCT has a good discriminatory ability in the diagnosis of paediatric sepsis.

Furthermore, the current study documented a significant difference in the AUC among the different age groups with higher value of 0.899 in the neonatal age group. The higher value recorded in neonates may be attributed to the high proportion of neonates with culture – proven sepsis and elevated PCT levels compared to other age groups in the current study. In a systematic review and meta-analysis conducted by Rees et al.^24^ in low- and middle-income countries, an AUC of 0.87 (95% CI 0.70 to 0.92) was reported also in neonates which is comparable to the value obtained in this present study. In contrast, studies by Arowosegbe et al 18 and Ezeh et al.^23^ reported an AUC of 0.686 and 0.520 respectively at a lower cutoff of 0.5 ng/mL. This AUC values were lower than those obtained in this current study. They may be due to the semiquantitative PCT assay method used by Arowosegbe et al 18 and the conventional blood culture method used by Ezeh et al.^23^ respectively.

Interestingly, children with gram – negative septicaemia had higher PCT levels than those with gram – positive septicaemia (4.68 ng/mL versus 2.14 ng/mL) in this current study. The ability of gram – negative bacteria to induce higher levels of procalcitonin was also documented by Bassetti et al.^25^ and Castillo-Bejarano et al 26 in Italy and Spain respectively and this was reported as statistically significant. Similarly, Nath et al.^21^ also recorded a higher PCT values in children with gram – negative septicaemia than those with gram – positive septicaemia (19.12 ng/mL versus 0.54 ng/mL) although the significance of this difference was not evaluated by the researchers.

## Conclusion

Procalcitonin is a reliable biomarker for diagnosing bacterial sepsis in children and can enhance clinical decision-making in resource-limited settings. Its integration into diagnostic protocols should be considered to improve outcomes. This study has demonstrated that serum PCT possesses considerable diagnostic value as a biomarker for bacterial sepsis among children aged 0–59 months in a tertiary healthcare setting in South Eastern Nigeria. The sensitivity of 66.9% and specificity of 89.7% at a PCT cut-off of ≥2.0 ng/mL, together with a positive predictive value (85.6%) and negative predictive value (74.9%), underscore its reliability as an adjunct diagnostic tool alongside blood culture. Notably, the area under the receiver operating characteristic curve (AUC) of 0.80 further affirms PCT's good discriminative power, which is particularly pronounced in neonates (AUC 0.899).

In a context where diagnostic delay from conventional blood cultures remains a challenge, and empirical antibiotic use contributes to escalating antimicrobial resistance, the integration of PCT testing could facilitate timelier clinical decision-making and more judicious antibiotic stewardship especially in low- and middle-income countries.

Nevertheless, while PCT testing cannot wholly supplant blood cultures—which remain the definitive standard for pathogen identification and antibiotic susceptibility testing—its role as a complementary marker is clear. This study therefore recommends the adoption of PCT assays, where feasible, into standard diagnostic protocols for paediatric sepsis in resource-constrained settings such as Nigeria. Additionally, further multicentre studies involving larger paediatric cohorts across diverse geographical regions are advocated to refine optimal cut-off values and to explore cost-effectiveness, especially in settings with varying burdens of infectious diseases and laboratory capacity.

## Figures and Tables

**Figure 2:**
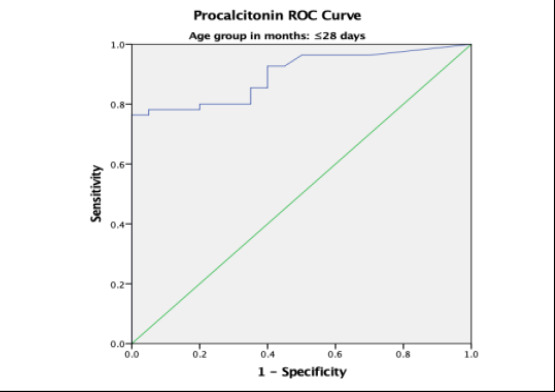
Procalcitonin ROC in neonatal age group

**Figure 3:**
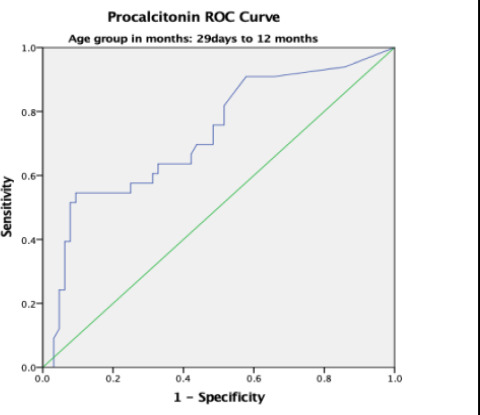
Figure. Procalcitonin ROC in 29 days to 12 months-age group

**Figure 4:**
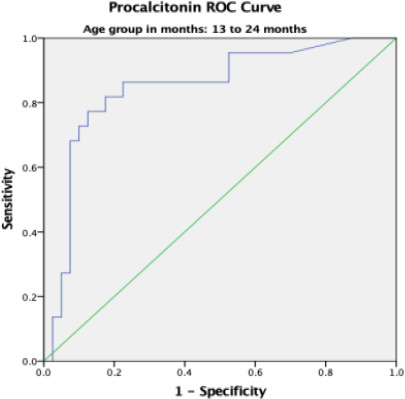
Procalcitonin ROC in 13 months to 24 months-age group

**Figure 5:**
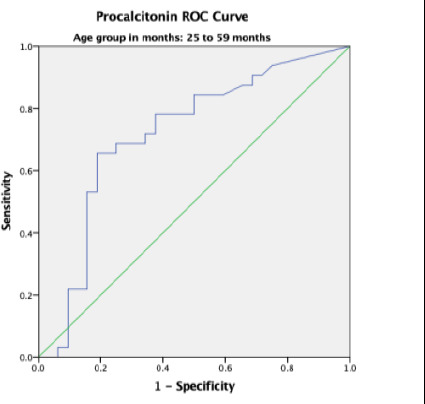
Procalcitonin ROC in 25 to 59 months-age group

**Figure 6:**
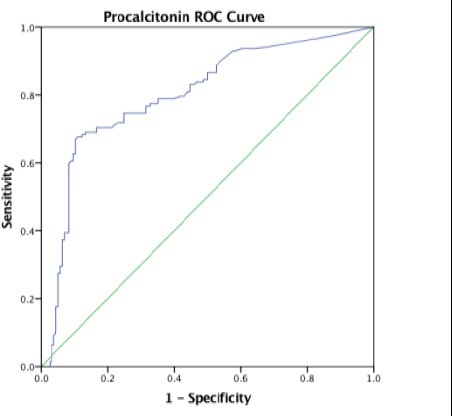
Procalcitonin ROC across all age groups
